# Depressive symptoms are associated with daytime sleepiness and subjective sleep quality in dementia with Lewy bodies

**DOI:** 10.1002/gps.4389

**Published:** 2015-11-11

**Authors:** Greg J. Elder, Sean J. Colloby, Debra J. Lett, John T. O'Brien, Kirstie N. Anderson, David J. Burn, Ian G. McKeith, John‐Paul Taylor

**Affiliations:** ^1^ Institute of Neuroscience, Campus for Ageing and VitalityNewcastle UniversityNewcastle upon TyneUK; ^2^ Department of Psychiatry, Cambridge Biomedical CampusUniversity of CambridgeCambridgeUK

**Keywords:** dementia with Lewy bodies, sleep quality, daytime sleepiness, depression, depressive symptoms

## Abstract

**Objective:**

Sleep problems and depression are common symptoms in dementia with Lewy bodies (DLB), where patients typically experience subjectively poor sleep quality, fatigue and excessive daytime sleepiness. However, whilst sleep disturbances have been linked to depression, this relationship has not received much attention in DLB. The present cross‐sectional study addresses this by examining whether depressive symptoms are specifically associated with subjective sleep quality and daytime sleepiness in DLB, and by examining other contributory factors.

**Methods:**

DLB patients (*n* = 32) completed the Pittsburgh Sleep Quality Index (PSQI), Epworth Sleepiness Scale (ESS) and the 15‐item Geriatric Depression Scale (GDS‐15). Motor and cognitive functioning was also assessed. Pearson correlations were used to assess the relationship between GDS‐15, ESS and PSQI scores.

**Results:**

GDS‐15 scores were positively associated with both ESS (*r* = 0.51, *p* < 0.01) and PSQI (*r* = 0.59, *p* < 0.001) scores.

**Conclusions:**

Subjective poor sleep and daytime sleepiness were associated with depressive symptoms in DLB. Given the cross‐sectional nature of the present study, the directionality of this relationship cannot be determined, although this association did not appear to be mediated by sleep quality or daytime sleepiness. Nevertheless, these findings have clinical relevance; daytime sleepiness or poor sleep quality might indicate depression in DLB, and subsequent work should examine whether the treatment of depression can reduce excessive daytime sleepiness and improve sleep quality in DLB patients. Alternatively, more rigorous screening for sleep problems in DLB might assist the treatment of depression. © 2015 The Authors. *International Journal of Geriatric Psychiatry* published by John Wiley & Sons, Ltd.

## Introduction

Dementia with Lewy bodies (DLB) is a common cause of dementia and accounts for 15–20% of all dementia cases post‐mortem (Holmes *et al.*, 1999). Individuals with DLB display a range of symptoms, which include motor and neuropsychiatric problems, attentional, executive function and visuospatial deficits, autonomic dysfunction and fluctuating levels of alertness, which is a key feature of DLB (McKeith *et al.,* 2005; McKeith, 2006).

Sleep disturbances are extremely common in individuals with DLB, where the main sleep problem is REM behaviour disorder (RBD), which refers to the inability to maintain muscle atonia during REM sleep. This symptom has been investigated in detail: RBD can predate the occurrence of Lewy body disease, is associated with various DLB clinical and pathological features and may have a prevalence of up to 70% in this condition (Ferman *et al.*, 2004; Dugger *et al.*, 2012; Iranzo *et al.*, 2013; Murray *et al.*, 2013; Ferman *et al.*, 2014). Other sleep disturbances, such as poor subjective sleep quality, are also prevalent in DLB, and are more severe than those observed in patients with Alzheimer's disease (AD), despite similar levels of impairment (Ferman *et al.*, 2014). In particular, daytime sleepiness is a very common and troublesome feature of DLB (Boot *et al.*, 2013).

The aetiology of non‐RBD sleep disturbances are currently poorly understood in DLB. For example, with regards to poor sleep quality, it is unclear whether this is because of the presence of concurrent neuropsychiatric symptoms such as hallucinations and agitation; both of which can cause nocturnal sleep disturbances, or whether this is because of the concurrent use of psychotropic medication (Bliwise *et al.*, 2011). Although large‐scale studies of the prevalence of non‐RBD sleep disorders in DLB are currently lacking, one in‐hospital study of 29 patients indicated that sleep apnoea and restless legs are both common symptoms in DLB, with frequencies of 30.7% and 50% respectively (Terzaghi *et al.*, 2013). Also of relevance are nocturnal motor symptoms, which are particularly common in those with parkinsonism (Grandas and Iranzo, 2004), although it is not known whether the severity of motor symptoms can affect sleep quality in DLB. Similarly, despite the high prevalence of excessive daytime sleepiness in DLB, it is unclear whether there is a direct link between poor sleep quality and daytime sleepiness in this population. Consequently, there is a lack of well‐evidenced treatments for these symptoms in DLB.

One potential risk factor for sleep disruption in DLB is the presence of depressive symptoms. Depression is a common symptom in patients with DLB, with an estimated prevalence of up to 60% (Fritze *et al.*, 2011). The relationship between depressive symptoms and sleep is well‐characterised in non‐dementia populations. Large‐scale studies in older adults have observed associations between depression and poor sleep; in a normative sample of 1506 older men and women in the United States (aged 55–84 years), individuals with depressive symptoms displayed a higher probability of experiencing several insomnia symptoms, and daytime sleepiness, compared to individuals without depressive symptoms (Foley *et al.*, 2004). Similarly, one cross‐sectional study of 3051 men (aged 67 years and older) showed that depressive symptoms, as measured using the 15‐item Geriatric Depression Scale (GDS‐15), were strongly associated with subjective sleep disturbances, specifically poor sleep quality and daytime sleepiness (Paudel *et al.,* 2008).

As the aetiology of subjectively poor sleep quality and daytime sleepiness, and their relationship with depressive symptoms, has yet to be established in DLB, the aim of this cross‐sectional study was to investigate the association between depressive symptoms, subjective sleep quality and daytime sleepiness in DLB patients.

## Methods

### Participants

A total of 55 patients and their informants were approached to take part in the study from dementia and movement disorder clinics in the North East of England (Newcastle upon Tyne and Sunderland). To be included in the study, participants were required to have a diagnosis of DLB and a reliable informant (e.g. carer or family member) who could assist with the completion of study assessments. A total of 10 patients declined to participate in the study. Participants were recruited sequentially in accordance with DLB diagnostic criteria (McKeith, 2006), and the diagnosis was confirmed by two experienced clinicians (JOB and IGM). Participants and their informants provided written informed consent, and the study was approved by the local NHS Research Ethics Committee.

### Procedure

Participants, with the aid of a reliable informant and in the presence of a researcher, completed measures of subjective sleep quality, daytime sleepiness and depression. Informants were, in the majority of participants, a spouse and/or a bed partner.

Subjective sleep quality was determined using the Pittsburgh Sleep Quality Index (PSQI; Buysse *et al.*, 1989), a seven‐item questionnaire which assesses self‐reported sleep disturbances over the past month. A total score of between zero and 21 is derived from the PSQI; a cut‐off value of five differentiates good and poor sleepers and higher scores indicate poorer sleep quality. The PSQI was also used to ask whether snoring, long pauses between breaths, behaviours indicative of RBD, or twitching or jerking legs occurred during sleep in the previous month. Subjective daytime sleepiness was assessed using the Epworth Sleepiness Scale (ESS; Johns, 1991), where higher scores reflect a greater degree of daytime sleepiness and a score of more than 10 reflects excessive daytime sleepiness. Depression was assessed using the GDS‐15 (Yesavage *et al.*, 1982), a yes/no questionnaire where scores of between zero and 15 are obtained. Higher scores represent more depressive symptoms, and scores of more than five indicate probable depression (D'Ath *et al.*, 1994). Notably, the GDS‐15 does not contain any questions which specifically ask about sleep quality or daytime sleepiness.

Cognitive function was also assessed using the Mini‐Mental State Examination (MMSE; Folstein *et al.*, 1975) and the Cambridge Cognitive Examination (CAMCOG; Roth *et al.*, 1986). Cognitive fluctuations were assessed using the Clinical Assessment of Fluctuation scale (CAF; Walker *et al.*, 2000). Extrapyramidal motor function was assessed using Part III of the Unified Parkinson's Disease Rating Scale (UPDRS‐III; Goetz *et al.,* 2008). Information regarding the duration of cognitive and parkinsonian symptoms, and the equivalent levodopa dose of anti‐parkinsonian medication, expressed in milligrams (mg) was also collected. Participant body mass index was calculated on the basis of objectively measured height and weight data (weight(kg) / height^2^(m)).

### Statistical analysis

The diagnosis of DLB was subsequently confirmed neuropathologically at post‐mortem for nine participants. Two participants were shown to have a diagnosis other than DLB following neuropathological assessment. Seven participants provided incomplete GDS‐15, ESS or PSQI assessment data, and four participants were unable to complete the assessments following consent. These participants (*n* = 13) were subsequently excluded from further analyses.

The primary aim of the current study was to investigate the association between depressive symptoms and sleep quality and daytime sleepiness. Secondary analyses examined whether there were relationships between demographic variables (age, the durations of cognitive and parkinsonian symptoms), clinical measures (MMSE, CAMCOG, CAF and UPDRS‐III scores) and sleep quality and daytime sleepiness assessment scores (ESS and PSQI), using Pearson correlations. PSQI scores were also used to classify participants as ‘good’ (scores of 5 or below) or ‘poor’ (scores > 5) sleepers in order to examine differences in GDS‐15 and ESS scores, using *t*‐tests. Similarly, ESS scores were used to classify participants as not excessively sleepy (indicated by ESS scores of 10 or below) or excessively sleepy (ESS scores > 10), and differences in GDS‐15 and PSQI scores were examined using *t*‐tests.

As a secondary analysis, GDS‐15, ESS and PSQI scores were compared between DLB patients who were, and who were not, taking antidepressant medication (*n* = 6), cholinesterase inhibitors (*n* = 10), levodopa medications (*n* = 6) or sleeping medications (*n* = 9), using *t*‐tests or Mann–Whitney *U* tests, where appropriate. Only two participants were taking atypical antipsychotic medications (risperidone and quetiapine), and these data were not analysed further.

## Results

Complete GDS‐15, PSQI and ESS data were obtained from a final sample of 32 participants (M_age_ = 76.16 years, SD_age_ = 7.03 years), 18 of which (56.3%) had bed partners. Demographic and clinical results are shown in Table 1, and overall GDS‐15, PSQI and ESS scores indicated poor subjective sleep, excessive daytime sleepiness and probable depression in the group of DLB patients. A total of 21 participants (65.6%) had a GDS‐15 score of >5, 13 participants (40.6%) had PSQI scores of >5 and 19 participants (59.4%) had an ESS score of >10. Bed partners reported that in the previous month, eight participants (25%) experienced snoring, six participants (18.75%) displayed behaviours indicative of RBD, two participants (6.25%) experienced long pauses between breaths during sleep and 10 (31.3%) participants experienced twitching or jerking legs during sleep.

**Table 1:** Participant demographic and clinical features (*n* = 32)

	Mean	SD
Age	76.16	7.03
Gender (male/female)	18 M (56.3%)/14 F (43.8%)	
Duration of cognitive symptoms (years)	2.60	1.99
Duration of parkinsonian symptoms (years)	1.77	1.38
Body mass index (kg/m^2^)	25.39	3.15
GDS‐15	5.66	3.45
PSQI	5.28	3.77
ESS	11.66	6.08
MMSE	17.66	4.88
CAMCOG	64.27	10.94
UPDRS‐III	29.09	15.29
Levodopa equivalent dose (mg)^a^	225.00	133.23

CAMCOG, Cambridge Cognitive Examination; ESS, Epworth Sleepiness Scale, GDS‐15, Geriatric Depression Scale (15‐item), MMSE, Mini‐Mental State Examination; PSQI, Pittsburgh Sleep Quality Index; UPDRS‐III, Part III of the Unified Parkinson's Disease Rating Scale.

a *n* = 6.

No demographic or clinical variables (including the levodopa equivalent dose; range 50–400 mg) were significantly associated with either ESS or PSQI scores. Additionally, ESS and PSQI scores were not significantly related (*r* = 0.33, *p* > 0.05). Primary analyses indicated that GDS‐15 scores were significantly positively associated with ESS scores (*r* = 0.51, *p* < 0.01) and that GDS‐15 scores were significantly positively associated with PSQI scores (*r* = 0.59, *p* < 0.001), demonstrating that more severe depressive symptoms were associated with excessive daytime sleepiness and poorer sleep quality. These relationships are summarised in Table 2. When patients were separated into ‘good’ (*n* = 19) or ‘poor’ sleepers (*n* = 13) on the basis of PSQI scores, poor sleepers displayed significantly higher GDS‐15 scores (*t*(30) = −3.63, *p* < 0.01) but not ESS scores (*t*(30) = −1.41, *p* > 0.05) than good sleepers. When patients were separated into those who were not excessively sleepy (*n* = 13) and those who were excessively sleepy (*n* = 19), on the basis of ESS scores, those who were excessively sleepy had significantly higher GDS‐15 scores (*t*(29.53) = −3.62, *p* < 0.01), but not PSQI scores (*t*(30) = −1.63, *p* > 0.05).

**Table 2:** Pearson correlations between measures of depressive symptoms, daytime sleepiness and sleep quality (*n* = 34)

	GDS‐15	ESS	PSQI
GDS‐15	—		
ESS	0.51*	—	
PSQI	0.33	0.59**	—

Notes:

ESS, Epworth Sleepiness Scale, GDS‐15, Geriatric Depression Scale (15‐item); PSQI, Pittsburgh Sleep Quality Index.

* *p* < 0.01.

** *p* < 0.001.

There were no significant differences in ESS, PSQI or GDS‐15 scores between those who were, and were not, currently taking antidepressant medication or cholinesterase inhibitors (all *p*‐values > 0.05). Participants who were taking medications containing levodopa showed significantly higher PSQI scores compared to those who were not, indicating worse sleep quality (*U =* 36, *p* < 0.05), but did not show any differences in ESS or GDS‐15 scores. Participants who were taking sleeping medications had significantly higher ESS (*U* = 53.5, *p* < 0.05), PSQI (*U* = 53, *p* < 0.05) and GDS‐15 scores (*U* = 37.5, *p* < 0.01), indicating a greater degree of excessive daytime sleepiness, worse sleep quality and greater depressive symptoms.

## Discussion

This study examined the association between depression and subjective sleep quality and daytime sleepiness in individuals with DLB. These results indicate that depressive symptoms are associated with both subjective sleep quality and daytime sleepiness in DLB. However, disease severity, expressed in terms of cognitive function or disease duration, was unrelated to sleep quality or daytime sleepiness. Similarly, motor symptom severity was not directly associated with either sleep quality or daytime sleepiness. This suggests that excessive daytime sleepiness and poor sleep quality in DLB may be driven more by depressive symptoms than cognitive or motor symptoms. This finding is in agreement with a previous study conducted in individuals with DLB, which observed that the severity of motor symptoms, and objective sleep quality in the preceding night, were not primarily responsible for daytime sleepiness (Ferman *et al.*, 2014).

Daytime sleepiness and sleep quality were also unrelated to the presence or severity of cognitive fluctuations in the present study. It is possible that this is because of the choice of fluctuation scale used, as one study has observed a relationship between informant‐rated fluctuations and daytime sleepiness, on the basis of the Mayo Fluctuations Scale (Ferman *et al.*, 2014). However, this study also objectively assessed daytime sleepiness on the basis of a multiple sleep latency test, and notably Ferman and colleagues found that there was no association between fluctuations and objective sleepiness. In addition, a separate study did not observe a relationship between objective intra‐individual measures of cognitive fluctuation, and intra‐individual measures of objective alertness, as determined by a maintenance of wakefulness test (Bliwise *et al.,* 2014), and thus it may be that cognitive fluctuations are not directly related to sleep or arousal.

Importantly, these results indicate that sleep problems are associated with depression, which is common in DLB (Fritze *et al.*, 2011), suggesting that excessive daytime sleepiness or poor sleep quality may be a sign of depression in DLB. Speculatively, these results are suggestive of a common aetiology linking depression and daytime sleepiness in DLB; one explanation may be that both are driven by deficits in the noradrenergic system. DLB patients exhibit a loss of noradrenergic neurons in the locus coeruleus which may have impacts on arousal, cognition and mood (Szot *et al.*, 2006; Benarroch, 2009). As norepinephrine can directly or indirectly contribute to the wake‐promoting effects of certain medications (Mitchell and Weinshenker, 2010), treatment options for daytime sleepiness in DLB may therefore include noradrenergic agents. Certainly, there is some evidence to suggest that drugs such as modafinil or admodafinil may be beneficial for this purpose (Boeve *et al.*, 2012) and it would be relevant to explore whether these agents can also improve mood. Whilst it is a possibility that sleep and depression are related to the same biological substrate, it is possible that the presence of depressive symptoms might cause sleep difficulties in DLB and that the screening and treatment of depressive symptoms might improve sleep. Alternatively, sleep difficulties may be the cause of depressive symptoms in DLB. As both possibilities have important treatment implications in DLB, these should be investigated in more detail.

Specific strengths of the current study include the well‐characterised nature of the patient cohort, as the diagnosis of DLB was made by two highly experienced clinicians, and neuropathological confirmation for the diagnosis was available for nine participants. In addition, the associations between daytime sleepiness and depressive symptoms, and sleep quality and depressive symptoms, were not because of the influence of cholinesterase inhibitors. A small number of patients who were taking sleep medications unsurprisingly tended to have poorer sleep quality, as well as excessive daytime somnolence. Similarly, patients who were taking medications containing levodopa (as a marker of greater disease severity) had poorer sleep quality.

Limitations of the current study include the associative nature of the findings and the lack of objective sleep data in this group of patients, as the data were cross‐sectional. However, this study was preliminary in nature and is the first to confirm the association between depressive symptoms, sleep quality, and daytime sleepiness, in individuals with DLB. As there is a well‐established association between depression and sleep in non‐dementia groups (Foley *et al.*, 2004; Paudel *et al.*, 2008), it is possible that a bi‐directional relationship between depressive symptoms and sleep quality, and between depressive symptoms and daytime sleepiness, exists in DLB. Future research should examine whether the severity of depressive symptoms, or concurrent neuropsychiatric symptoms such as nocturnal visual hallucinations, which are common in DLB, can influence the degree of daytime sleepiness, or sleep quality, in DLB. A longitudinal study would clarify the directionality of this relationship and confirm whether depression precedes daytime sleepiness and poor sleep quality in DLB. This has been shown in one non‐DLB community study in a South Korean sample of adults over 65 years of age (Kim *et al.*, 2009). Larger comparative studies may also be useful in identifying whether the relationships between sleep and depressive symptoms are more severe in DLB than in other dementias, or healthy aged individuals. Additionally, future studies may also wish to employ the use of sleep diaries, from which more detailed sleep continuity data can be derived, thus allowing the relationship between specific aspects of subjective sleep quality and depressive symptoms to be examined in greater detail.

The examination of sleep and depression in DLB may be particularly salient in early or prodromal disease stages, as both sleep and depressive symptoms may antedate the manifestation of dementia by many years (Donaghy *et al.,* 2015). Additionally, future research should further clarify the reliability and validity of commonly used sleep measures, such as the PSQI and ESS, in dementia populations. Finally, it is possible that the presence of sleep disordered breathing, restless legs syndrome (RLS) or periodic limb movements (PLMs) may have affected sleep in the DLB group we examined, although there was no reason to expect these participants should show different rates to those previously reported (Terzaghi *et al.*, 2013). However, a detailed large‐scale assessment of the prevalence of RLS or PLMs, and the subsequent effects upon sleep, has yet to be conducted in this patient group (Iranzo *et al.*, 2007).

## Conclusions

Depressive symptoms are associated with subjectively worse sleep quality and excessive daytime sleepiness in individuals with DLB. These results potentially indicate that the treatment of depressive symptoms may result in corresponding improvements to subjective sleep quality and daytime sleepiness in DLB. Excessive daytime sleepiness or poor sleep quality may also be a sign of depression in DLB, and might suggest that more rigorous screening for depression is warranted in DLB in order to aid the treatment of sleep difficulties.

## Conflict of interest

The authors report no conflicts of interest.

Key points

- Poor sleep quality and excessive daytime sleepiness are common in DLB.
- Poor sleep quality and daytime sleepiness are associated with depressive symptoms.
- The treatment of depressive symptoms may improve sleep, and daytime sleepiness, in DLB.
- Potential bi‐directional relationships between sleep and mood in DLB need further examination.
